# Development of Molecularly Imprinted Magnetic Amino Acid-Based Nanoparticles for Voltammetric Analysis of Lead Ions in Honey

**DOI:** 10.3390/polym16131782

**Published:** 2024-06-24

**Authors:** Mehmet Karagözlü, Süleyman Aşır, Nemah Abu Shama, Ilgım Göktürk, Fatma Yılmaz, Deniz Türkmen, Adil Denizli, Murat Özgören

**Affiliations:** 1 Department of Food Engineering, Faculty of Agriculture, Near East University, Nicosia 99138, Cyprus; mehmet.karagozlu@neu.edu.tr; 2 Research Center for Science, Technology and Engineering (BILTEM), Near East University, Nicosia 99138, Cyprus; 3 Department of Biomedical Engineering, Faculty of Engineering, Near East University, Nicosia 99138, Cyprus; 4 Department of Analytical Chemistry, Faculty of Pharmacy, Near East University, Nicosia 99138, Cyprus; 5 Department of Chemistry, Hacettepe University Ankara, Ankara 06800, Turkey; 6 Department of Chemistry and Chemical Processing Technologies, Bolu Abant Izzet Baysal University, Bolu 14030, Turkey; yilmaz_f@ibu.edu.tr; 7 Department of Biophysics, Faculty of Medicine, Near East University, Nicosia 99138, Cyprus; murat.ozgoren@neu.edu.tr

**Keywords:** lead, imprinted polymer, magnetic nanoparticles, carbon paste electrode, electrochemical biosensor, differential pulse voltammetry, honey

## Abstract

Lead (Pb) is a hazardous metal that poses a significant threat to both the environment and human health. The presence of Pb in food products such as honey can pose a significant risk to human health and is therefore important to detect and monitor. In this study, we propose a voltammetric detection method using molecularly imprinted polymer (MIP) electrodes to detect Pb (II) ions in honey. Pb (II) ion-imprinted amino acid-based nanoparticles with magnetic properties on a carbon paste electrode (MIP-CPE) were designed to have high sensitivity and selectivity towards Pb (II) ions in the honey sample. Zetasizer measurements, electron spin resonance, and scanning electron microscopy were used to characterize magnetic polymeric nanoparticles. The results showed that the voltammetric detection method using MIP-CPE was able to accurately detect Pb (II) ions in honey samples with a low detection limit. The proposed method offers a simple, rapid, cost-effective solution for detecting Pb (II) ions in honey. It could potentially be applied to other food products to ensure their safety for human consumption. The MIP-CPE sensor was designed to have high sensitivity and selectivity towards Pb (II) ions in the honey sample. The results showed that the technique was able to deliver highly sensitive results since seven different concentrations were prepared and detected to obtain an R^2^ of 0.9954, in addition to a low detection limit (LOD) of 0.0912 µM and a low quantification limit (LOQ) of 0.276 µM. Importantly, the analysis revealed no trace of Pb (II) ions in the honey samples obtained from Cyprus.

## 1. Introduction

If heavy metals such as lead (Pb), cadmium (Cd), arsenic, and mercury are present in food and water, they can be extremely dangerous to human health [1]. Pb exposure can cause various health complications, including damage to the nervous system, digestive issues, and developmental problems in children. The acceptable daily intake (ADI) of Pb is currently set at 0.05 milligrams per kilogram of body weight [2]. Recent studies have shown that even low-level Pb exposure can have adverse health effects, particularly in children [3]. The World Health Organization and the Centers for Disease Control and Prevention both recognize the dangers of Pb exposure and recommend efforts to reduce it [4]. Recently, several studies have been conducted to understand further the dangers of Pb exposure and its effects on human health [5,6,7]. Some of these analyses have focused on the effects of low-level Pb exposure, which was previously thought to be relatively safe. For example, a study published in JAMA Pediatrics in 2020 found that even low levels of Pb in the blood were associated with lower IQ scores and other cognitive problems in children [8]. Other recent studies have looked at the impact of Pb exposure on pregnancy outcomes and the development of chronic diseases such as cardiovascular disease [9].

Heavy metals are released into the soil, water, and air in significant amounts by transportation, industrial and agricultural operations, and other anthropogenic activities. Consequently, environmental heavy metal concentrations rise sharply and have an impact on both human health and the ecological balance [10]. Plants from the water, soil, and air can absorb heavy metals present in the environment. This process is known as bioaccumulation. As plants grow, they accumulate these metals in their tissues [11]. The region where the bees forage encompasses around 7 km² and has a variety of environments, vegetation, and food sources. Bees come into contact with plants, air, soil, and water when searching such enormous areas for nectar, pollen, honeydew, and plant exudates [12]. When honeybees forage for nectar and pollen from flowers, they may inadvertently collect heavy metals from the contaminated plants. Bees are essential pollinators, and they play a crucial role in transferring these contaminants through the food web. The process of honey production by bees involves collecting nectar from flowers and converting it into honey within the hive. If the nectar is sourced from plants in contaminated areas, heavy metals present in the nectar can be transferred to the honey. Consequently, honey can become contaminated with heavy metals. The levels of contamination can vary depending on the proximity of the bee colonies to pollution sources, the types of plants visited by the bees and water sources around the area that bees consume [11]. According to Codex Alimentarius, the maximum limit of Pb (II) ions in honey is 0.1 mg/kg [13].

For many years, researchers in different countries around the world have used conventional techniques such as atomic absorption spectroscopy (AAS) [14], inductively coupled plasma optical emission spectroscopy (ICP-OES) [15], inductively coupled plasma atomic emission spectroscopy (ICP-AES) [16], inductively coupled plasma mass spectroscopy (ICP-MS) [17], etc., for the detection of heavy metals in honey. Although these techniques are quite selective and sensitive, there are many difficulties in their use in heavy metal detection, such as high cost, long detection time, complex operational procedures, and challenges in obtaining detection in real environments. In order to overcome these drawbacks, electrochemical methods (ECMs) have been widely accepted in heavy metal detection in many recent studies. These ECMs provide the same sensitivity with shorter detection times, lower cost, and less complex operational procedures [18]. The electrochemical technique is a compact system consisting of two or three main electrode sensors, depending on the program and method that is undertaken. One of these electrodes is a working electrode (WE), designed to interact selectively with the analyte. Various types of WEs are employed in the electrochemical sensors, such as carbon paste electrodes (CPEs), glassy carbon electrodes, gold electrodes, etc. Many modifications have recently been applied to these types of WEs to increase the sensitivity and performance of the analysis, for example, using nanoparticles or polymers [19]. Molecularly imprinted polymers (MIPs) are an important example of the polymers used to modify the WE for a specific target [20]. In the presence of the target molecule, functional and cross-linking polymerization creates structure, size, and stereo-specific cavities. The template is then removed from the cavities as mentioned above, allowing the target to be available for rebinding [21]. Hence, the electrochemical sensors provide quick, selective, and sensitive recognition behavior towards the target substance when combined with MIPs [22].

Nanoparticles, due to their small sizes, offer several benefits in the field of electrochemical sensing by expanding the electrode’s surface area. Additionally, they can provide a quick electron transfer and raise the mass-transport rate, both of which increase the sensitivity of the electrodes being employed [18]. Some commonly used nanoparticles are silver nanoparticles, gold nanoparticles, bismuth nanoparticles, and magnetic nanoparticles (mNPs). Because of their unique magnetic properties and simplicity in surface functionalization, mNPs, like iron oxides, have become increasingly important in electrochemical biosensors. Target analytes may be easily separated and enriched from complicated samples, and remote manipulation utilizing external magnetic fields is made possible by the use of mNPs [23]. This makes it easier to construct quick and accurate biosensing systems. mNPs modified with MIPs have shown promise as electrochemical biosensor materials. MIPs provide target analytes with particular molecular recognition sites. The controlled immobilization of the molecules and the extraction of analytes from samples are made possible by their integration with mNPs [24]. In this research, the novelty involves developing a biosensor using Pb (II) ion-imprinted amino acid-based magnetic polymeric nanoparticles on a carbon paste electrode (MIP-CPE). The purpose of the biosensor is to determine the presence of the Pb ions that are likely to be present in honey via fast, easy, cost-effective, and environmentally friendly electrochemical analysis by using differential pulse voltammetry (DPV) method.

## 2. Materials and Methods

### 2.1. Materials and Reagents

L-Cysteine methyl ester hydrochloride (C_4_H_9_NO_2_S HCl, 98%) was obtained from Across Organics (Fair Lawn, NJ, USA). Methacryloyl chloride and magnetic nanopowder (Fe_3_O_4_) were supplied by Aldrich (Milwaukee, WI, USA). The chemicals used to prepare a buffer to obtain a pH of 1.5 were sodium dihydrogen phosphate adjusted with phosphoric acid; acetate buffers (ABS) to obtain a pH of 4.8 and 5.5 were acetic acid and sodium acetate; phosphate buffers to obtain a pH of 7.0 and 7.4 were potassium phosphate monobasic and potassium phosphate dibasic; the buffer to obtain a pH of 8.5 was ammonium chloride adjusted by ammonia; and lastly, to obtain a pH of 10.5, carbonic acid was adjusted by sodium hydroxide. All of those chemicals, as well as nitric acid and hydrochloric acid, were supplied by Merck (Darmstadt, Germany). The deionized water (18.2 MΩ cm) used for buffer solutions was supported by Purelab Ultra Analytic (ELGA Lab Water, Runcorn, UK). Pb (II) nitrate and Cd nitrate tetrahydrate were obtained from Sigma-Aldrich. The stock solution of Pb (II) ions (100 µM) was prepared by dissolving the calculated quantity of Pb (II) nitrate in 0.1 M nitric acid solution. Stock solution of Cd (II) ions (50 µM) was prepared by dissolving the proper amount of cadmium nitrate tetrahydrate in 0.1 M nitric acid solution. Honey was obtained from the local beekeepers of Cyprus and kept in the dark at ambient temperature until the analysis. Tombow 0.5 mm HB pencil tips were purchased from the local store. Paraffin oil was obtained from Doğa İlaç Hammaddeleri (Istanbul, Turkey).

### 2.2. Apparatus

Cyclic voltammetry (CV) and DPV measurements were performed by the AUTOLAB-PGSTAT204 potentiostat/galvanostat with the NOVA 2.1.2 software package (Metrohm, Utrecht, The Netherlands). A three-electrode cell was utilized for electrochemical analysis. The first electrode was a reference electrode composed of Ag/AgCl with 3 M KCl. The second electrode was platinum wire used as a counter electrode. The third electrode was the working electrode. Pb (II) ion-imprinted amino acid-based magnetic polymeric nanoparticles on a carbon paste electrode (MIP-CPE), non-imprinted amino acid-based magnetic polymeric nanoparticles on a carbon paste electrode (NIP-CPE), and bare carbon paste electrode (CPE) were used for this purpose. All electrochemical experiments were carried out at ambient temperature (25 ± 1 °C). Upper vertex potential, lower vertex potential, number of scans, and the scan rate for the CV method were +0.5 V, −1.5 V, 1, and 0.05 V/s, respectively. For the DPV method, the parameters of start potential, stop potential, step height, and pulse amplitude were −1.0 V, +0.0 V, 0.005 mV, and 0.025 V, respectively. 

### 2.3. Treatment of Honey Samples

To acquire the acid-treated honey solution, 100 mL of 0.1 M nitric acid was added to a beaker that contained 5 g of honey and stirred until reaching a clear yellow color without forming any precipitate. Then, 50 mL of the prepared acid-treated honey solution and Pb (II) nitrate were used to obtain a honey solution, which contains 100 μM Pb (II) ions. Before measurements were performed, this solution was spiked into the acid-treated honey samples in different proportions (from 5 μM to 100 μM). DPV was performed for both acid-treated honey samples and Pb (II) ion-spiked honey samples.

### 2.4. Synthesis of 2-Methacryloyl-Amido Cysteine (MAC) Functional Monomer

Molecular imprinting technique creating recognition sites in synthetic materials mimics chemical processes occurring in the biological systems. When ion-imprinted polymers are prepared, functional monomers containing electron-donating atoms such as S, N, or O are carefully considered for metal recognition. The present study synthesized a functional MAC monomer with thiol groups interacting with Pb (II) ions. The method used for synthesizing the monomer MAC was adapted from the procedure documented in the literature [25]. L-cysteine methyl ester hydrochloride (5.0 g) and NaNO_2_ (0.2 g) were dissolved in 30 mL of a 5% (*v*/*v*) K_2_CO_3_ aqueous solution. This solution was then cooled to 0 °C. Then, methacryloyl chloride (7.0 mL) was slowly added to the solution under a nitrogen atmosphere, followed by magnetic stirring at room temperature for 2 h. After the reaction’s completion, the solution’s pH was adjusted to 4.0, and it was extracted with ethyl acetate. The aqueous phase was evaporated using a rotary evaporator, and the resulting residue (MAC) was crystallized in a mixture of diisopropyl ether and ethyl acetate.

### 2.5. Pb (II) Ion-Imprinted Amino Acid-Based Magnetic Polymeric Nanoparticles

Pb (II) ion-imprinted amino acid-based magnetic polymeric nanoparticles (MIP-mNPs) were developed for the specific detection of Pb (II) ions in honey samples. The selection of the complexing monomer is based on the strong attraction of side chain sulfhydryl groups within the functional monomer MAC toward Pb (II) ions. In a standard ion imprinting procedure, the functional monomer MAC was first combined with the template ion Pb (II) before the polymerization process, forming a noncovalent coordination complex. In a typical ion imprinting process, the functional monomer MAC was complexed with the template ion Pb (II) ions prior to the polymerization process to form a noncovalent coordination complex.

MIP-mNPs were synthesized by the mini-emulsion polymerization method. Firstly, 0.2 mmol of MAC monomer in 10 mL of pH 7.0 phosphate buffer was mixed with 0.1 mmol of Pb(NO_3_)_2_ for 60 min at room temperature to form a metal-chelated monomer. Then, 0.1 g of sodium dodecyl sulfate as surfactant and 0.1 g of polyvinyl alcohol (PVA) as stabilizer were also dissolved in 20 mL of water, and Fe_3_O_4_ was added to the mixture to obtain the first phase. Later, 0.03 g of sodium dodecyl sulfate and 0.2 g PVA were dissolved in 10 mL of water to obtain the second phase. NaHCO_3_ (0.025 g) was utilized to adjust the pH of the water. After 200 μL of the metal-chelated monomer was added into the monomer mixture consisting of 2 mL of ethylene glycol dimethacrylate (EGDMA) and 1 mL of 2-hydroxyethyl methacrylate (HEMA), the mixture was poured in the second phase and homogenized at 5000 rpm for 20 min. The emulsion was moved to the neck glass balloon containing the first phase in the water bath. Then, 100 mg of ammonium persulfate (APS) and 50 mg of NaHSO_3_ were added to the final solution and mixed at 600 rpm for 1 day at 40 °C. MIP-mNPs were centrifugated with ethanol at 30,000 rpm for 60 min several times to remove any unreacted monomer. After washing with 0.5 M NaOH for 24 h for the desorption of Pb (II) ions from the MIP-mNPs, it was washed with deionized water for 24 h. The same method was used to synthesize non-imprinted magnetic polymeric nanoparticles (NIP-mNPs); however, Pb (II) ions were not added to the monomer mixture. In addition, Pb (II) ion-imprinted amino acid-based non-magnetic polymeric nanoparticles (MIP-NPs) were prepared using the same procedure except for adding Fe_3_O_4_. The polymeric nanoparticles were dried by lyophilization before use. 

### 2.6. Preparation of CPE Sensors 

To prepare the MIP-CPE sensor, 60 mg of graphite powder was obtained by grinding the 0.5 mm HB Tombow pencil tips and mixing with 15 mg of MIP-mNPs. After that, 25 mg of paraffin oil was added to that mixture and mixed for 10 min until a smooth wetted paste had been obtained. The prepared paste was transferred to the carbon paste electrode holder, and then the surface was smoothed by polishing it on the weighing paper. The same method was used to create non-imprinted amino acid-based magnetic polymeric nanoparticles on a carbon paste electrode (NIP-CPE). In contrast, non-imprinted magnetic nanoparticles were added to the mixture. CPE sensor was also prepared by just using graphite powder and paraffin oil. 

### 2.7. Characterization Studies

The size distribution of the magnetic polymeric nanoparticles was determined using dynamic light scattering (DLS, NanoS, Malvern Instruments, London, UK) (n = 3). Using electron spin resonance (ESR) spectroscopy, the magnetic properties of the nanoparticles were investigated in the 1000–5000 G magnetic field range by ESR analyzer. So, using the ESR method, the uptake of the magnetite nanoparticles into the polymeric structure was monitored. (Bruker EMX 113X-Band). MIP-mNPs and MIP-CPE sensors were also characterized by scanning electron microscopy (SEM, JSM-6400, JEOL, Akishima, Tokyo, Japan) to evaluate surface morphology after the samples were coated with a thin gold–palladium alloy coating. Surface characterization of MIP-CPE and NIP-CPE sensors was carried out by taking ten measurements from different parts of the surface using the sessile drop method with the Kruss DSA100 contact angle device (Hamburg, Germany). In addition, the MIP-mNPs and NIP-mNPs were characterized using a Fourier transform infrared spectrophotometer with an attenuated total reflection spectrophotometer (FTIR-ATR, Thermo Fisher Scientific, Nicolet iS10, Waltham, MA, USA), (wavenumber range of 400–4000 cm^−1^).

## 3. Results and Discussion

### 3.1. Characterization Studies

**Figure 1:**
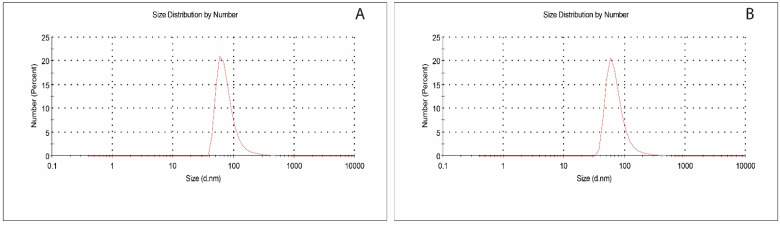
DLS analysis of magnetic polymeric nanoparticles (**A**) MIP-mNPs and (**B**) NIP-mNPs. Vertical axes denote number as percentage and horizontal axes denote size of particles.

The average size of the magnetic polymeric nanoparticles measured by DLS analysis demonstrated the hydrodynamic diameter of monodisperse particles with a uniform average nanoparticle size distribution, and the result was given in Figure 1. The average size of MIP-mNPs and NIP-mNPs was 80.27 nm (PDI:0.403) and 75.91 nm (PDI:0.469), respectively. As deduced from the results, the incorporation of Pb (II) ions increased the hydrodynamic size of the MIP-mNPs.

**Figure 2:**
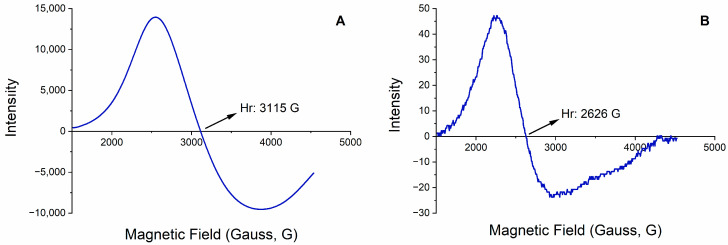
ESR results of magnetic (**A**) MIP-mNPs and (**B**) MIP-NPs polymeric nanoparticles. Vertical axes denote intensity and horizontal axes denote magnetic field (Gauss, G). Arrows indicate resonant magnetic field.

The ESR spectrum was obtained using the first derivative of the absorption curve by adjusting the magnetic field, as illustrated in Figure 2. The magnetic field’s intensity causes a shift in signal during the spectrum. The signal decreases after reaching its peak and returns to the zero point. Gauss (G) value was the resonance magnetic field (Hr) value for the magnetic polymeric nanoparticles. ESR spectroscopy confirmed that magnetic particles were present in the structure. So, ESR spectra were evaluated in the presence of Fe_3_O_4_ nanoparticles, and the magnetic field densities were calculated as 3115 G and 2626 G, respectively, for the synthesized MIP-mNPs and MIP-NPs. The G factor is described as a property of a molecule that has unpaired electrons. High-spin complexes were observed in the literature for Fe_3_O_4_ when the g factor fell within the range of 2.0 to 9.7, whereas low-spin complexes were detected within the range of 1.4 to 3.2 [26].

**Figure 3:**
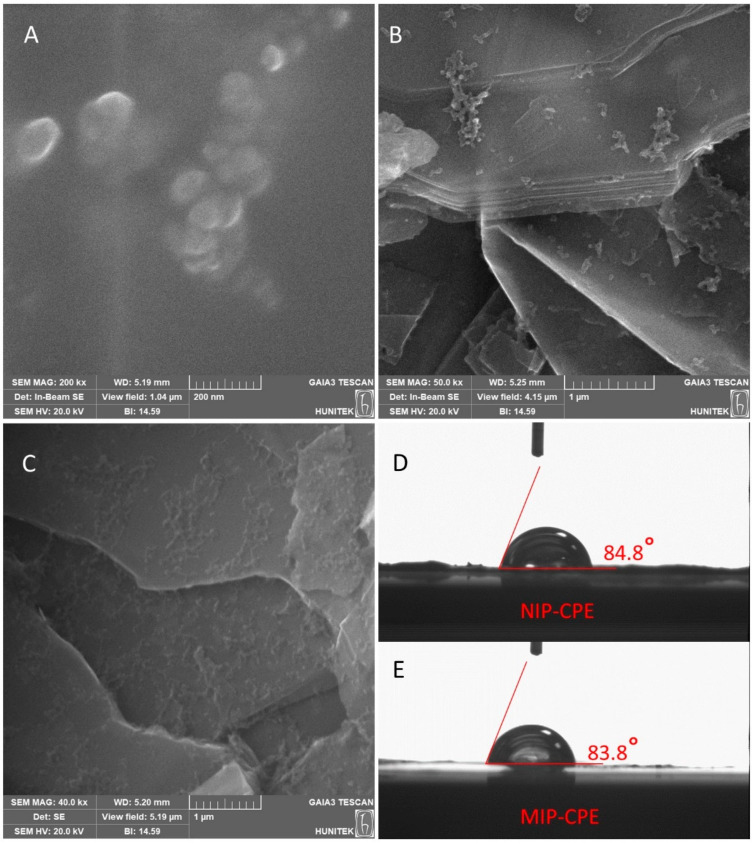
SEM images of (**A**) MIP-mNPs, (**B**) MIP-CPE sensor, (**C**) NIP-CPE sensor, (**D**) contact angle image of NIP-CPE sensor, and (**E**) contact angle image of MIP-CPE sensor.

In Figure 3, the surface morphology of both polymeric magnetic nanoparticles and CPE sensors was characterized by SEM. As shown in Figure 3A, MIP-mNPs have a non-porous and spherical structure. All nanoparticles were generated through the mini-emulsion polymerization, exhibiting a spherical shape with an approximate diameter of 100 nm. Thus, imprinting with Pb (II) ions does not cause a significant change in particle sizes. Figure 3B refers to the SEM image of the MIP-CPE sensor, while Figure 3C refers to the NIP-CPE sensor. Figure 3D,E represent the contact angle images of the NIP-CPE and MIP-CPE sensors, respectively. The approximated contact angle value for the NIP-CPE sensor was 84.8° ± 1.24, while the MIP-CPE sensor was 83.8° ± 0.97. The hydrophobicity of the MIP-CPE sensor surface decreases due to the Pb (II) ions coordination to MAC by molecular imprinting, so we observe that the contact angle of NIP-CPE is enhanced slightly compared to the MIP-CPE sensor.

**Figure 4:**
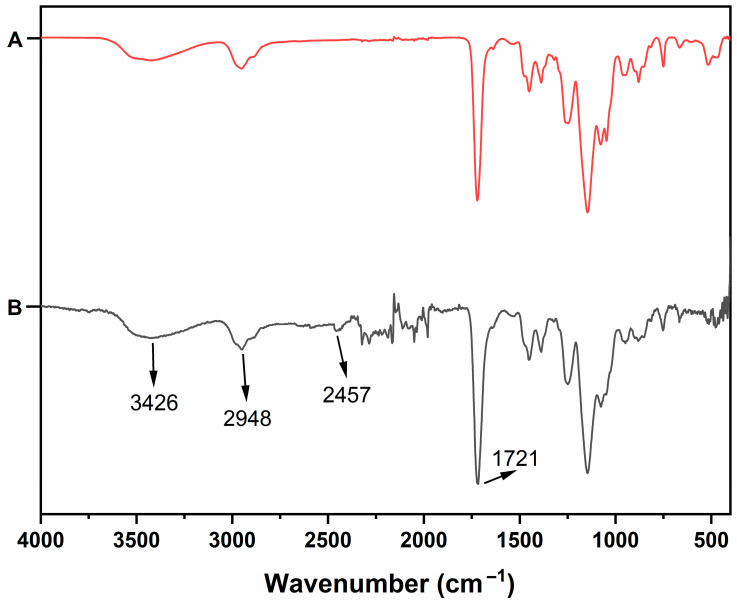
FTIR spectra of (**A**) NIP-mNPs (red line) and (**B**) MIP-mNPs (black line). Specified wavenumbers are shown with arrows.

As shown in Figure 4, the IR spectrum of the MIP-mNPs indicated that a polymeric layer has formed on the surface of the nanoparticles. O-H and C-H bonds exist by the peaks at 3426 and 2948 cm^−1^, respectively. These bonds are most likely because of the MAC and HEMA monomers in the polymeric structure. Furthermore, the existence of these monomers in the polymeric layer is further supported by the signal at 1721 cm^−1^, which represents the stretching vibration of C=O bonds. The peak at 2457 cm^−1^, representing the stretching vibration of Pb (II) ion-coordinated S-H bonds, disappears in the non-imprinted nanoparticles. The change could result from the cysteine monomer’s thiol groups attaching to the magnetic nanoparticles in the absence of Pb (II) ions.

### 3.2. Optimization of MIP-CPE Sensor

In order to optimize the medium for analysis of Pb (II) ions, different acids (HCl and Nitric acid) and buffer solutions that have different pHs (pH, 1.5, 4.8, 5.5, 7, 7.4, 8.5, and 10.5) were used. CV has very low sensitivity compared to DPV. To obtain the results, the concentration of the Pb (II) ions was increased from 100 µM to 100 mM in 100 mL. Lead nitrate did not dissolve in pH 8.5 and 10.5 buffer solutions, and precipitates were observed due to high concentration. It was noticed that 0.1 M HNO_3_ and ABS (pH 4.8 and 5.5) were the best solvents for Pb (II) ions analyte. 

**Figure 5:**
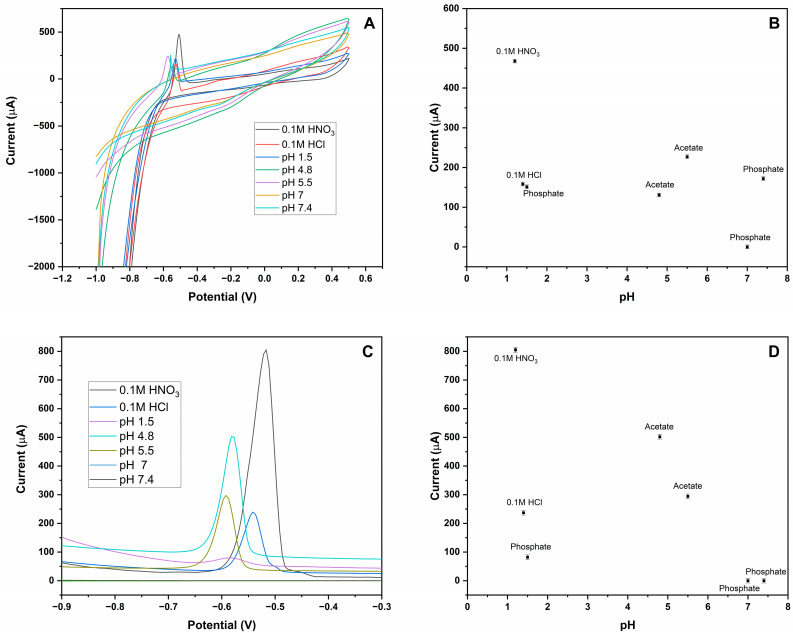
Optimization of the working pH for 100 mM Pb (II) ions by voltammetric method in different acids and buffer solutions at room temperature by MIP-CPE sensor. (**A**) Voltammograms of Pb (II) ions by CV in different acids and pH buffer solutions; (**B**) current (vertical axis) vs. pH (horizontal axis) graph of the CV responses; (**C**) voltammograms of Pb (II) ions by DPV in different acids and pH buffer solutions; (**D**) current (vertical axis) vs. pH (horizontal axis) graph of the DPV responses.

After preparing the Pb (II) ions stock solutions in different acid and buffer solutions, CV and DPV analyses were performed, respectively. The analysis of Pb (II) ions at different acid and buffer solutions, followed by the response of each voltammetric technique used with MIP-CPE as WE in the electrochemical cell, is shown in Figure 5. The maximum peak current was at 0.1 M HNO_3_ in CV and DPV. Therefore, 0.1 M HNO_3_ was selected and used in subsequent electrochemical measurements as a supporting electrolyte.

### 3.3. Analytical Performance of MIP-CPE Sensor

**Figure 6:**
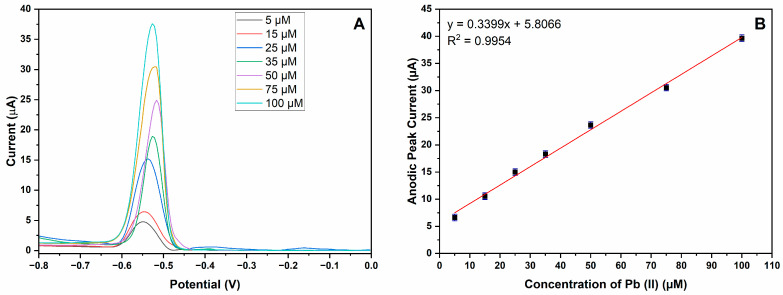
Analytical performance of MIP-CPE. (**A**) Voltammograms of Pb (II) ions by DPV via MIP-CPE sensor. Color code map in relation to µM is provided in the upper right corner; (**B**) calibration graph (vertical axis indicates anodic peak current and horizontal axis indicates concentration of Pb (II)) of Pb (II) ions in concentrations ranges from 5 µM to 100 µM. Red line indicates linear trend line.

The analytical performance of the MIP-CPE sensor for detecting Pb (II) ions was evaluated by DPV. Figure 6A shows the DPV response of Pb (II) ions with different concentrations at the MIP-CPE. Polymer coating on the magnetic nanoparticles is designed to create specific binding sites for the analyte, which brings specificity together with increased adsorption on the surface of electrode and which may cause a shift in the peak potential. As seen from Figure 6, the peak potential shifts from −0.55 V to −0.53 V as the concentration of Pb (II) ions increases. The adsorption of Pb (II) ions on the electrode surface is responsible for the shift to a higher potential observed with an increase in Pb (II) concentration. The degree of adsorption increases with concentration, changing the electrode’s surface characteristics and the potential needed for the redox reaction to occur. As predicted, an acceptable linear relationship was observed with the peak currents as the concentrations of target metal ions increased from 5 to 100 μM. The linear regression equation for Pb (II) ion is calibrated as *I*_Pb_ (current/μA) = 5.8066 + 0.3399*C*_Pb_ (concentration/μM) (*C*_Pb_: 5–100 μM) with a correlation coefficient (R^2^) of 0.9954, as shown in Figure 6B. The limit of detection (LOD) is calculated as 3.3 times the standard deviation divided by the slope of the calibration curve, and the limit of quantification (LOQ) is calculated as 10 times the standard deviation divided by the slope of the calibration curve. The LOD and LOQ of the developed electrode were 0.0912 μM and 0.276 μM, respectively.

### 3.4. Estimation of Selectivity and Imprinting Efficiency

**Figure 7:**
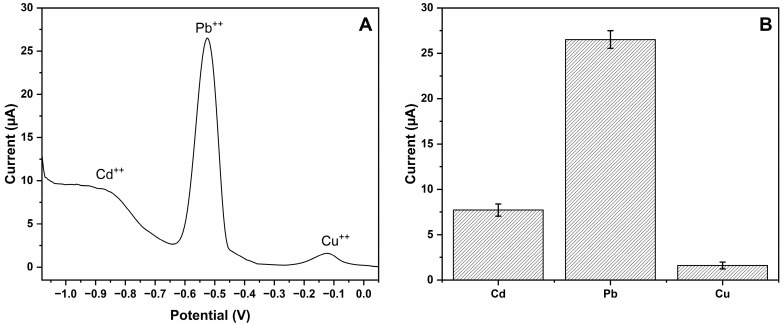
Selectivity study. (**A**) MIP-CPE sensor response to Pb (II) ion (50 µM), Cd (II) ion (50 µM), and Cu (II) ion (50 µM) competitors; (**B**) column representation of the MIP-CPE sensor responses (n = 3) with error bars.

To determine the selectivity of the MIP-CPE sensor towards Pb (II) ions, competing studies were conducted in the presence of Cd (II) ions and Cu (II) ions, as shown in Figure 7. Selectivity assessment for Pb (II) ions was performed by applying competitor ions, namely Cd (II) ions and Cu (II) ions, in the same solution as Pb (II) ions. The concentration for Pb (II) ions, Cd (II) ions, and Cu (II) ions was 50 µM. Compared to competitor ions Cd (II) and Cu (II) ions, the developed MIP-CPE sensor exhibited 3.44 and 16.47 times greater selectivity in detecting the target Pb (II) ions than the competing ion, respectively. The utilization of the imprinting technique resulted in the formation of template-shaped cavities within the polymer matrices, demonstrating predetermined selectivity and a strong affinity for the detection of Pb (II) ions.

**Figure 8:**
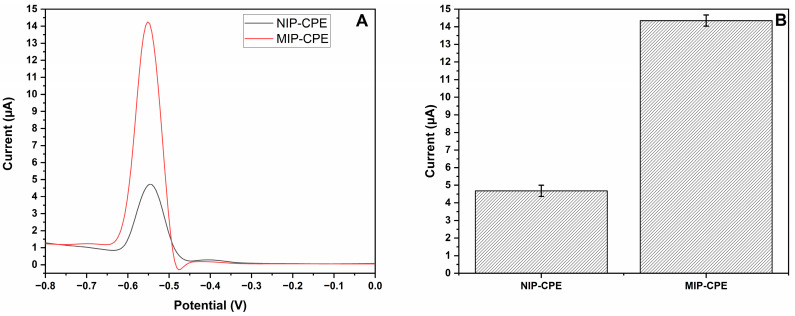
The response of the modified electrodes. (**A**) MIP-CPE (red line) and NIP-CPE (black line) sensor responses for 25 µM of Pb (II) ions; (**B**) column representation of the MIP-CPE and NIP-CPE sensor responses (n = 3) with error bars.

**Figure 9:**
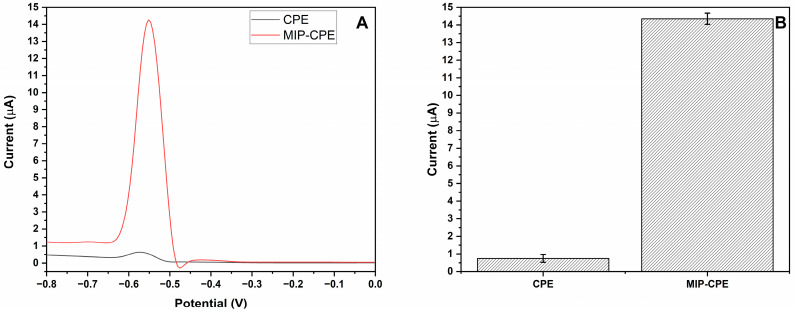
The response of the modified electrodes. (**A**) MIP-CPE (red line) and CPE (black line) sensor responses for 25 µM of Pb (II) ions; (**B**) column representation of the MIP-CPE and CPE sensor responses (n = 3) with error bars.

In order to assess the imprinting efficiency of the MIP-CPE sensor, the imprinting factor was determined by comparing the Pb (II) ions current peak response produced by MIP-CPE sensor signal with the NIP-CPE sensor (Figure 8) and CPE sensor (Figure 9) signals. It was determined that the MIP-CPE sensor was 3.07 and 19.13 times more selective for Pb (II) ions when compared to NIP-CPE and CPE sensors, respectively.

### 3.5. Comparison of MIP-CPE Sensor with Previous Studies

Table 1 compares the produced MIP-CPE sensor with the previously voltammetric Pb (II) sensors reported in the literature, including the method, supporting electrolyte correlation ratio linear range, and LOD. As can be seen, the LOD values obtained in the present work are higher than those obtained with other electrodes. In some studies, screen-printed electrodes modified with single-walled carbon nanohorns and bismuth film [27] (Yao et al., 2019) and fluorescent carbon dots and gold nanoparticles [28] observed lower LOD values than those in the current study. Also, in a study in which a glassy carbon electrode with a modified polymer [29] had been used obtained a lower LOD value than that in this study. It was due to using different types of electrodes and different modifying agents. In addition, some studies that used CPE with various materials to modify the CPE such as bismuth [30] and europium [31] demonstrated lower LOD values than those in this study. However, the application conditions and the cost of the sensors should also be considered. For example, carbon paste electrodes are cheaper than screen-printed electrodes and glassy carbon electrodes. Additionally, the materials used for modifying the electrodes, such as bismuth, europium, and gold nanoparticles, are more expensive than the materials used in this study. Also, square wave anodic stripping voltammetry (SWASV) methods demonstrated better sensitivity with the LOD in the nM range.

### 3.6. Determination of Pb (II) Ions in Real Sample

**Figure 10:**
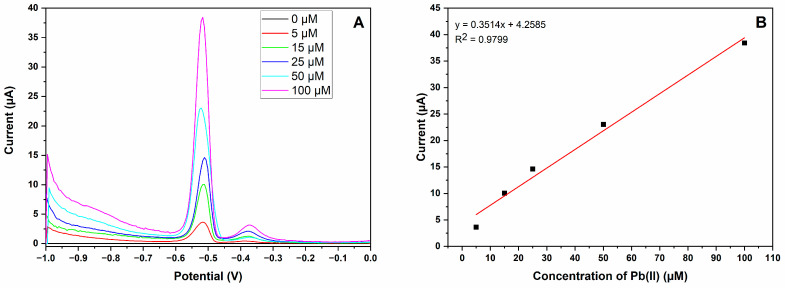
(**A**) Voltammograms of acid-treated honey samples containing Pb (II) ions in different concentration ranges from 0.0 µM to 100 µM by DPV using the MIP-CPE sensor. Color code map in relation to µM is provided in the upper right corner; (**B**) calibration graph (vertical axis indicates current and horizontal axis indicates concentration of Pb (II)) of Pb (II) ions in different concentration ranges from 5 to 100 µM. Red line indicates linear trend line.

The determination of Pb (II) ions in acid-treated honey samples was evaluated by DPV. Figure 10A illustrates the DPV response of Pb (II) ions with different concentrations in acid-treated honey solution at MIP-CPE. DPV responses of Pb (II) ions in acid-treated honey samples showed a similar trend with analytical performance. An acceptable linear relationship with the peak currents at the peak position (−5.1 V) of Pb (II) ions was observed, with the concentration of target metal ions increasing from 0 to 100 µM. The linear regression equation for Pb (II) ions in acid-treated honey solution is calibrated as *I*_Pb_ (current/μA) = 4.2585 + 0.3514*C*_Pb_ (concentration/μM) (*C*_Pb_: 5–100 μM) with an R^2^ of 0.9799, as shown in Figure 10B. The correlation coefficient of real samples was less than the analytical performance due to the matrix effect of the acid-treated honey solution. No response was observed at the peak position of Pb (II) ions when the honey sample was used without adding Pb (II) ions into the solution. Thus, the real sample demonstrates no Pb (II) ions. Additionally, Pb (II) ion analyses of honey samples were also examined using ICP-MS for verification purposes. The linear regression equation for the ICP-MS method is calibrated as y = 0.202 + 1.525*C*_Pb_ (C_Pb_: 1–100 ppb) with an R^2^ of 0.9988 and LOD of 0.0079 ppb, as shown in Figure S1. The result of the ICP-MS analysis of honey samples verified the result of the voltammogram.

## 4. Conclusions

Pb is a hazardous metal that poses a significant threat to both the environment and human health. Since Pb (II) ions in honey can be hazardous to human health, detecting and monitoring their presence is critical. A simple and efficient CPE modified with Pb (II) ion-imprinted amino acid-based magnetic nanoparticles were successfully fabricated for the analysis of Pb (II) ions. Zetasizer measurements, ESR, SEM, contact angle measurements, and FTIR were used for the characterization studies. The MIP-CPE sensor demonstrated a good analytical performance under optimal conditions for detecting Pb (II) ions, with an LOD of 0.0912 µM, LOQ of 0.276 µM, and R^2^ of 0.9954. The selectivity and imprinting efficiency were also studied. The MIP-CPE sensor exhibited 3.44- and 16.47-times-greater selectivity in detecting the target Pb (II) ions than the competing Cd (II) ions and Cu (II) ions, respectively. Also, it was 3.07 and 19.13 times more selective for Pb (II) ions when compared to NIP-CPE and CPE sensors, respectively. According to Codex Alimentarius, the maximum limit of the Pb (II) ions in the honey is 0.1 mg/kg (equivalent to 0.675 µM when the density of the honey is around 1.4 g/cm^3^), which is higher than our LOD value. The MIP-CPE sensor can be effectively utilized to determine the Pb (II) ions in honey samples.

## Figures and Tables

**Table 1:** Comparison of MIP-CPE sensor parameters (^a–c^) with previous studies.

Electrode	Method	Electrolyte	^a^ R^2^	^b^ LR (µM)	^c^ LOD (µM)	Reference
MIP-CPE	DPV	0.1 M HNO_3_	0.9954	5.0–100	0.0912 µM	This study
SPE/SWCNHs/BiF ^1^	SWASV	0.1 M ABS (pH 4.5)	0.9866	4.83 × 10^−3^–0.290 µM	1.93 × 10^−3^ µM	[27]
Eu^3+^-doped NiO/CPE ^2^	SWASV	0.1 M ABS (pH 4.5)	0.997	3.86 × 10^−3^–0.796 µM	4.83 × 10^−4^ µM	[31]
SPCE/CDs/AuNP ^3^	DPV	0.1 M PBS (pH 2.0)	0.964	0.0483–1.30 µM	0.02 µM	[28]
Pb(II)-IIP-GCE ^4^	DPV	0.2 M HAc-NaAc buffer (pH 4.5)	0.9997	0.05–60 µM	0.01 µM	[29]
Bi–Sb/CPE ^5^	SWASV	ABS (pH 5.6)	0.994	4.83 × 10^−3^–0.724 µM	1.40 × 10^−3^ µM	[30]
EDTA_PANI/SWCNTs/SS ^6^	DPV	0.5 M H_2_SO_4_	0.9897	2–37 µM	1.65 µM	[32]
AuNP-deposited porous carbon thread	DPV	HCl–KCl (pH 2)	0.979	10–110 µM	1.419 μM	[33]
PGMGPE ^7^	CV	0.1 M PBS (pH 4.5)	0.996	200–450 μM and 500–1200 μM	0.8 μM	[34]

^a^ Correlation coefficient, ^b^ linear range, ^c^ limit of detection. ^1^: Single-walled carbon nanohorn-modified screen-printed electrode with bismuth film; ^2^: CPE modified with europium-doped nickel oxide. ^3^: Screen-printed carbon electrode modified by fluorescent carbon dots and gold nanoparticles. ^4^: Pb-imprinted polymer (MAA-EGDMA with 8-hydroxyquinoline ligand) on Glassy carbon electrode. ^5^: Bismuth antimony nanocomposite-modified carbon paste electrode. ^6^: Polyaniline, single-walled carbon nanotubes, and ethylenediaminetetraacetic acid-modified stainless steel electrode. ^7^: Polyglycine-modified graphene paste electrode.

## Data Availability

The authors can confirm that all relevant data are included in the article.

## Supplementary Materials

The following supporting information can be downloaded at: https://www.mdpi.com/article/10.3390/polym16131782/s1, Figure S1: Calibration graph of Pb (II) by ICP-MS. Including linear regression equation (Eqn.), correlation coefficient (Cor.Coeff.), limit of detection (DL) and calibration table.
