# Supplementary material for: Development of Molecularly Imprinted Magnetic Amino Acid-Based Nanoparticles for Voltammetric Analysis of Lead Ions in Honey
Source: Polymers (Basel). 2024 Jun 24;16(13):1782. doi: 10.3390/polym16131782 (PMC11244471; doi:10.3390/polym16131782)
Supplement: Supplementary file 1 [file polymers-16-01782-s001.zip › polymers-3023802-supplementary.pdf]

Development of Molecularly Imprinted Magnetic Amino Acid-Based Nanoparticles for Voltammetric Analysis of Lead Ions in Honey

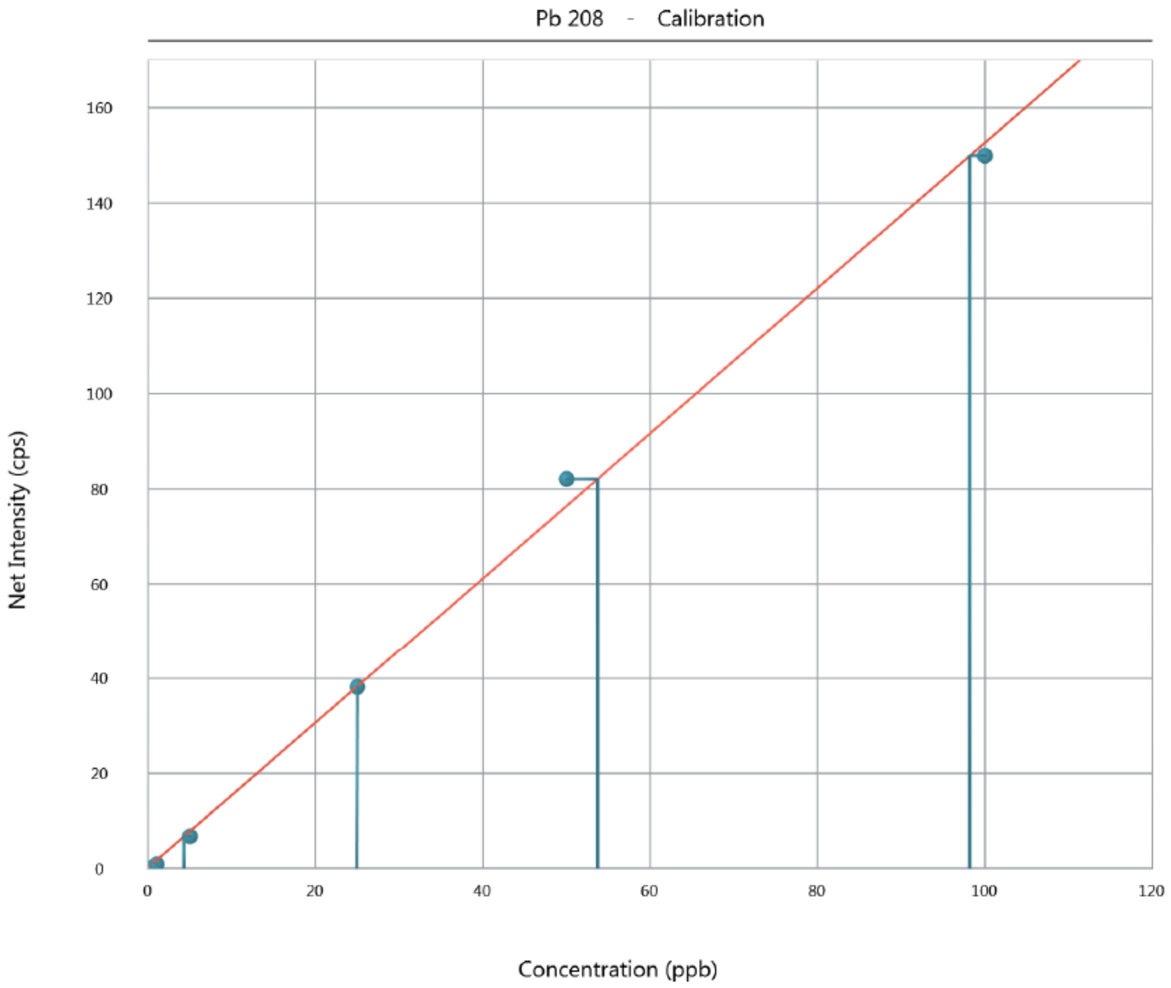

Statistics

Eqn. :	y = 1.525x + 0.202	
Cor.Coeff. :	0.998820	
BEC :	0.931523	ppb
DL :	0.007923	ppb

Calibration Table

	Net Intensity Pb 208 (cps)	Apparent Conc. Pb 208 (ppb)
Blank	15295.4	
Cal. Std.1	0.1	-0.081
Cal. Std.2	0.9	0.445
Cal. Std.3	6.8	4.301
Cal. Std.4	38.2	24.916
Cal. Std.5	82.1	53.709
Cal. Std.6	149.9	98.210

Figure S1. Calibration graph of Pb (II) by ICP-MS. Including linear regression equation (Eqn.), correlation coefficient (Cor.Coeff.), limit of detection (DL) and calibration table
